# YOLO-ACT: an adaptive cross-layer integration method for apple leaf disease detection

**DOI:** 10.3389/fpls.2024.1451078

**Published:** 2024-10-01

**Authors:** Silu Zhang, Jingzhe Wang, Kai Yang, Minglei Guan

**Affiliations:** ^1^ School of Computer Science and Software Engineering, University of Science and Technology Liaoning, Anshan, China; ^2^ Institute of Applied Artificial Intelligence of the Guangdong-Hong Kong-Macao Greater Bay Area, Shenzhen Polytechnic University, Shenzhen, China; ^3^ School of Artificial Intelligence, Shenzhen Polytechnic University, Shenzhen, China

**Keywords:** foliar disease, object detection, YOLOv8s, feature fusion, task-aligned, intelligent agriculture

## Abstract

Apple is a significant economic crop in China, and leaf diseases represent a major challenge to its growth and yield. To enhance the efficiency of disease detection, this paper proposes an Adaptive Cross-layer Integration Method for apple leaf disease detection. This approach, built upon the YOLOv8s architecture, incorporates three novel modules specifically designed to improve detection accuracy and mitigate the impact of environmental factors. Furthermore, the proposed method addresses challenges arising from large feature discrepancies and similar disease characteristics, ultimately improving the model's overall detection performance. Experimental results show that the proposed method achieves a mean Average Precision (mAP) of 85.1% for apple leaf disease detection, outperforming the latest state-of-the-art YOLOv10s model by 2.2%. Compared to the baseline, the method yields a 2.8% increase in mAP, with improvements of 5.1%, 3.3%, and 2% in Average Precision, Recall, and mAP50-95, respectively. This method demonstrates superiority over other classic detection algorithms. Notably, the model exhibits optimal performance in detecting Alternaria leaf spot, frog eye leaf spot, gray spot, powdery mildew, and rust, achieving mAPs of 84.3%, 90.4%, 80.8%, 75.7%, and 92.0%, respectively. These results highlight the model’s ability to significantly reduce false negatives and false positives, thereby enhancing both detection and localization of diseases. This research offers a new theoretical foundation and direction for future advancements in apple leaf disease detection.

## 1 Introduction

Apple leaf diseases are one of the primary factors affecting apple growth and yield (Jiang et al., 2019). Accurate and rapid identification of leaf diseases is crucial for farmers to take timely measures. Traditional diagnostic methods mainly rely on expert observation and analysis, such as judging diseases based on the color, size, and shape of leaf spots. These methods are not only time-consuming but also require a high level of expertise, making large-scale operations difficult.

In recent years, with the rapid development of artificial intelligence technology, object detection has become a popular research direction for apple leaf disease detection. Object detection technology locates and identifies objects in images, enabling classification and definition of the objects (Zhao et al., 2019). Typically, object detection algorithms are divided into two-stage and one-stage algorithms. Although two-stage algorithms are more accurate, they have limitations in computational cost and real-time performance. In contrast, one-stage algorithms are more suitable for resource-constrained environments due to their speed and efficiency (Kamath and Renuka, 2023). YOLO (You Only Look Once) is a one-stage object detection algorithm proposed by Redmon et al (Redmon et al., 2016). It achieves rapid object detection by using DarkNet, a convolutional neural network framework.

YOLOv8, introduced by Ultralytics in 2023, combines the advantages of previous versions and incorporates new backbone networks and detection heads, significantly improving detection speed and accuracy.YOLOv8 is divided into five models: n, s, m, l, and x, with increasing network depth and detection accuracy, suitable for various hardware platforms, including low-end mobile devices.

In this study, we trained and tested our model using the ALDD dataset, which was fusion from two open-source datasets. The dataset includes not only dense and small disease targets but also larger targets that affect the leaf veins and even entire leaves. Additionally, there are challenges related to similar features among diseases of similar sizes, which poses a significant test for the feature extraction capabilities of the model. Moreover, in mainstream methods, the detection head typically optimizes two subtasks: object classification and localization, using a dual-branch task approach. This often leads to unreasonable label assignment between the two tasks, retaining incorrect results and affecting detection performance.

To address these challenges, we propose a disease detection network based on YOLOv8s. Experiments conducted on images with complex backgrounds demonstrate that the proposed algorithm has practical value in apple cultivation and production. The main contributions and innovations of this work are summarized as follows:

1. We enhance the original Path Aggregation Network with Feature Pyramid Network (PAN-FPN) structure in YOLOv8s by adding an Adjacent Feature Fusion (AFF) module. This module strengthens cross-layer feature fusion and integrates shallow features into a small target detection layer to address the issue of significant target shape variation.
2. The C2f module in the neck of the network is replaced with a Cascade Attention Module (CAM) that employs iterative attention mechanisms. This enhancement improves feature extraction and fusion capabilities to tackle the problem of similar disease characteristics.
3. The traditional decoupled head is replaced with a Dynamic Tack-Aligned Head (DTAH), which enhances task alignment in the detector and increases interaction between classification and localization tasks. This approach guides the model to dynamically adjust its receptive fields while retaining results with high localization and confidence.
4. A comparative analysis of several typical object detection algorithms was conducted. Experimental results show that our algorithm achieves an mAP of 85.1% on the ALDD dataset. This algorithm provides support for precise planting, visual management, and intelligent decision-making in apple production.

The structure of this paper is as follows: Section 2 summarizes related work; Section 3 introduces the dataset composition and the YOLOv8s network; Section 4 presents the proposed apple leaf disease detection model; Section 5 details the experimental setup, including the experimental environment and evaluation metrics; Section 6 showcases and analyzes the experimental results; and Section 7 provides conclusions and suggestions for future research.

## 2 Related work

The field of fruit tree disease detection is similar to that of crop disease detection, with early research primarily relying on traditional image processing techniques and machine learning algorithms. With the rapid development of deep learning, convolutional neural networks (CNNs) have been increasingly applied in disease detection. Researchers have achieved significant detection results by constructing various CNN architectures and training them on large-scale datasets. Common deep learning models include AlexNet (Krizhevsky et al., 2017), VGG (Simonyan and Zisserman, 2014), and ResNet (He et al., 2016). Additionally, in the field of object detection, models such as Faster R-CNN (Ren et al., 2015), YOLO (Redmon et al., 2016), and SSD (Liu et al., 2016) have been widely used in fruit tree disease detection.

Due to the importance of detection efficiency and real-time performance, one-stage models have gradually become the focus. Among these, the YOLO series has received considerable attention for its performance in disease detection. Numerous studies have improved YOLO models to enhance detection accuracy and efficiency.

One example is the work by Yiweng Wang et al (Wang et al., 2022), who proposed the MGA-YOLO model for apple leaf disease detection by integrating the Ghost module and Convolutional Block Attention Module (CBAM) into the YOLOv5 network. They achieved an mAP of 89.3% with a model size of only 10.34MB. Weishi Xu et al. (Xu and Wang, 2023) introduced Mobilenet-V3’s basic blocks and utilized group convolution and depthwise convolution for downsampling, designing ALAD-YOLO, which significantly improved the accuracy of tea leaf disease detection while reducing computational costs. Another example is the work by Zhenyang Xue et al (Xue et al., 2023), who enhanced tea leaf disease detection performance by integrating self-attention and CBAM into YOLOv5 and replacing YOLOv5’s original modules with Receptive Field Blocks (RFB).

In the domain of tea leaf disease detection, Md. Janibul Alam Soeb et al. (Soeb et al., 2023) addressed the issue of sample scarcity through data augmentation methods. They compared various object detection and recognition networks, confirming that YOLOv7 outperformed others in detecting and recognizing tea leaf diseases in natural scene images. Similarly, Xiaoqiang Yang et al. (Yang and Guo, 2023) chose the YOLOv7 algorithm for detecting apple diseases, improving model accuracy using the DCNV3 module and enhancing downsampling through a combination of Space-to-Depth (SPD) and Depthwise Separable Convolution (DSConv), resulting in a 3.3% accuracy increase, a parameter reduction of 0.38M, and a 1.5 FFLOPS (Fused Floating Point Operations Per Second) computational reduction.

With the release of YOLOv8, researchers have shifted their focus to this latest object detection model. Houda Orchi et al. (Orchi et al., 2023) evaluated YOLOv8 for crop leaf disease detection, assessing its accuracy, recall, precision, F1 score, confusion matrix, Frames Per Second(FPS), inference time, and performance in terms of bounding box, classification, and distribution loss, proving YOLOv8’s feasibility and capability in crop leaf disease detection. Consequently, Rujia Li et al. (Li et al., 2024) conducted research on YOLOv8 for maize leaf disease detection. They designed GhostNet Triplet YOLOv8s by integrating a lightweight GhostNet structure into YOLOv8, achieving a 0.3% increase in mAP, a 50.2% reduction in model size, and a 43.1% reduction in FLOPs for maize leaf disease detection. These research findings indicate that the YOLO model has broad application prospects in detecting diseases on fruit tree leaves. However, in reality, the morphological characteristics of apple leaf diseases vary significantly. Although the YOLO series algorithms have achieved satisfactory results in agricultural disease detection, the accuracy of some existing lightweight networks still needs improvement when it comes to detecting apple leaf diseases.

## 3 Materials and methods

### 3.1 Dataset description

In the field of apple leaf disease detection, data plays a crucial role. We obtained two publicly available datasets from the internet: PlantDoc (Singh et al., 2020) and AppleLeaf9 (Yang et al., 2022). The PlantDoc dataset includes three classes of disease images related to apple leaves, captured under real-world conditions in cultivated fields. The authors utilized deep learning methods to classify and test the dataset, confirming the importance of complex background data in advancing disease detection towards practical applications. AppleLeaf9 is a combination of datasets from Plant Village (Hughes and Salathe, 2016), ATLDSD (Feng and Chao, 2022), PPCD 2020, and PPCD 2021 (Thapa et al., 2020). Guided by domain experts, the authors clearly classified apple leaves into nine disease categories, including healthy leaves. The dataset was also tested using the proposed EfficientNet-MG algorithm, achieving high accuracy. These studies demonstrate the reliability and separability of the categories in both datasets.

However, we found that while the PlantDoc dataset features apple leaf diseases with complex outdoor backgrounds, it contains relatively few categories. Conversely, the AppleLeaf9 dataset offers a larger number of categories but suffers from significant class imbalance in disease occurrences. This imbalance can bias the model towards frequently occurring categories, making it easier to detect these targets while potentially missing or incorrectly detecting low-frequency categories. Such biases can affect the overall mean average precision (mAP), leading to evaluation metrics that may not fully reflect the model’s performance across all categories.

To address these issues, we made manual adjustments by first removing low-quality images and those that did not match the complex outdoor backgrounds required. We then integrated images with complex backgrounds from the PlantDoc dataset into the corresponding categories. Finally, we randomly deleted images from overrepresented categories to balance the dataset with underrepresented ones as much as possible. We named the resulting dataset the ALDD dataset.

**Figure 1:**
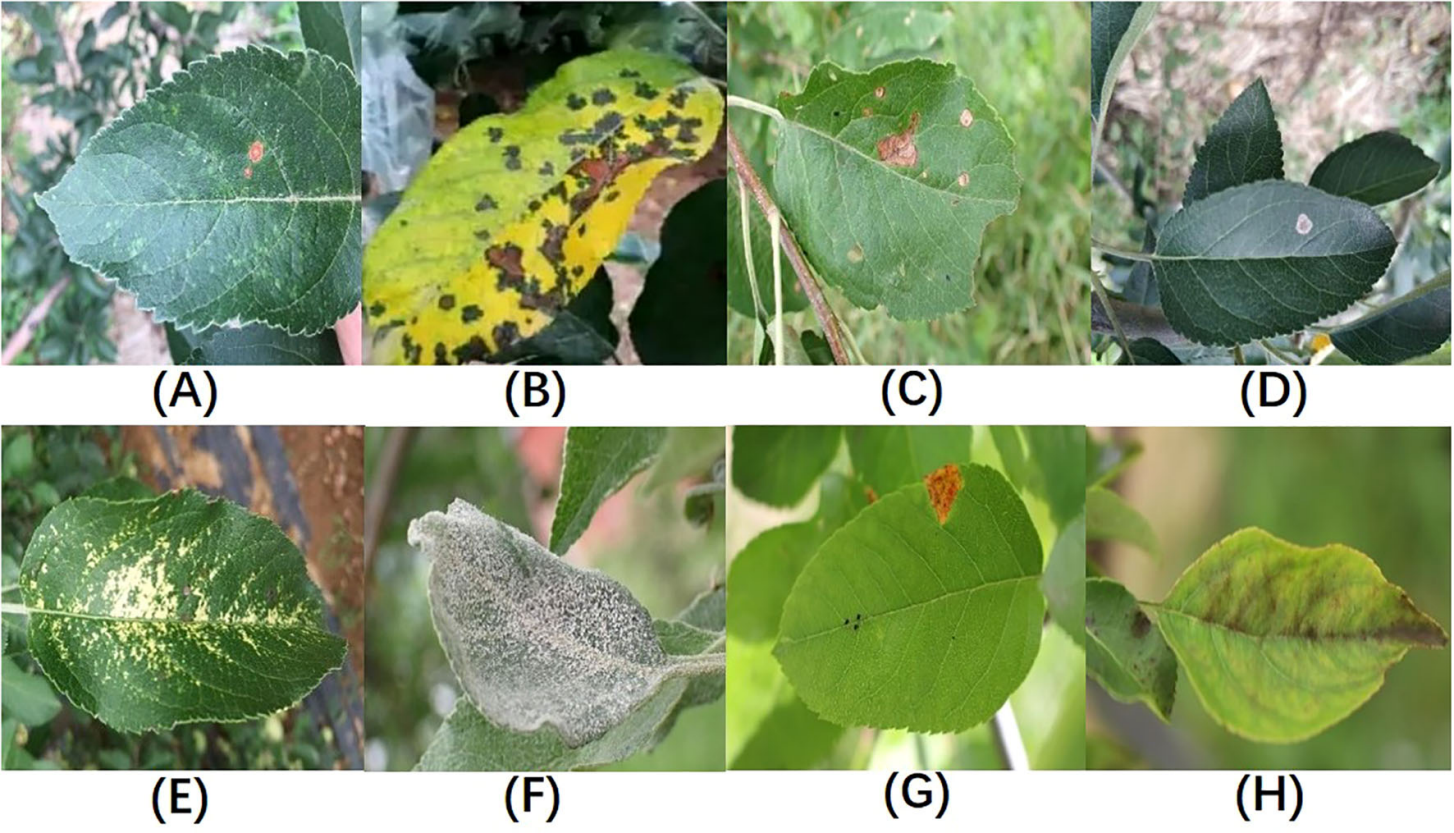
Sample Illustrations from the Dataset:**(A)** Alternaria leaf spot; **(B)** Brown spot; **(C)** Frogeye leaf spot; **(D)** Grey spot; **(E)** Mosaic disease; **(F)** Powdery mildew; **(G)** Rust; **(H)** Scab; Figure redrawn from (Yang et al., 2022).

In total, the dataset used in this study covers eight types of apple leaf diseases: Alternaria leaf spot, brown spot, frog-eye leaf spot, gray leaf spot, mosaic, powdery mildew, rust, and apple scab, with a total of 3,638 images of diseased leaves. The ALDD dataset presents several challenges for apple leaf disease detection in complex backgrounds: (1) Apple leaf disease images captured in real-world scenarios often include multiple leaves and complex backgrounds, such as branches and fruits, which can interfere with the localization of diseased areas; (2) Disease features are similar, and early-stage disease targets are small; (3) Lighting conditions vary with time of day. Images taken in the morning have weaker light, while those taken at noon can be overexposed with prominent shadows. These lighting issues may lead deep learning algorithms to mistakenly identify spots or shadows as disease features. **Figure 1** illustrates the various apple leaf diseases. Based on the descriptions and classifications of diseases in the dataset by Yang et al (Yang et al., 2022), and after discussions with domain experts, we have summarized the main symptoms and causes of apple leaf diseases, as shown in **Table 1**.

**Table 1:** Symptoms and etiology of eight apple leaf diseases.

Disease	Symptom	Etiology
Alternaria leaf spot	Early stages show circular or oval brown spots with distinct concentric rings or “target” appearance, accompanied by reddish-brown or purple edges. Later stages see spots enlarge and merge, leading to leaf yellowing, wilting, and even defoliation.	Alternaria Nees
Brown spot	Early stages show small brown spots with light red or purplish edges. In later stages, spots gradually enlarge, causing leaves to yellow, wilt, and even fall off.	Diplocarpon mali
Frogeye leaf spot	Early stages show small circular spots with a gray-white center and dark brown or purplish-brown edges, resembling “frog eyes.” Later stages see spots enlarge and merge, causing leaf yellowing, curling, and severe defoliation.	Cercospora sojina
Grey spot	Early stages show small gray-white spots with possible brown or reddish-brown edges. Later stages see spots enlarge and merge, causing large areas of leaf discoloration and even perforation.	Phyllosticta pirina
Mosaic	Early stages show mottled or striped patterns in light green, yellow-green, or white on leaves. Later stages may cause leaf deformation and curling.	Apple mosaic virus
Powdery mildew	Early stages show white powdery substances on the affected areas, with leaf edges curling upward and becoming erect. Later stages see black specks near leaf axils and main veins, potentially causing leaf shrinkage and premature defoliation.	Podosphaera leucotricha
Rust	Early stages show shiny orange-red small spots that gradually enlarge, forming circular orange-yellow lesions with red edges. In severe cases, a single leaf may have dozens of spots.	Gymnosporangium yamadai Miyabe
Scab	Early stages show light yellow-green circular or radial spots that gradually turn brown and eventually black. Infected leaves often show several merged spots.	Venturia inaequalis

### 3.2 YOLOv8s model description

**Figure 2:**
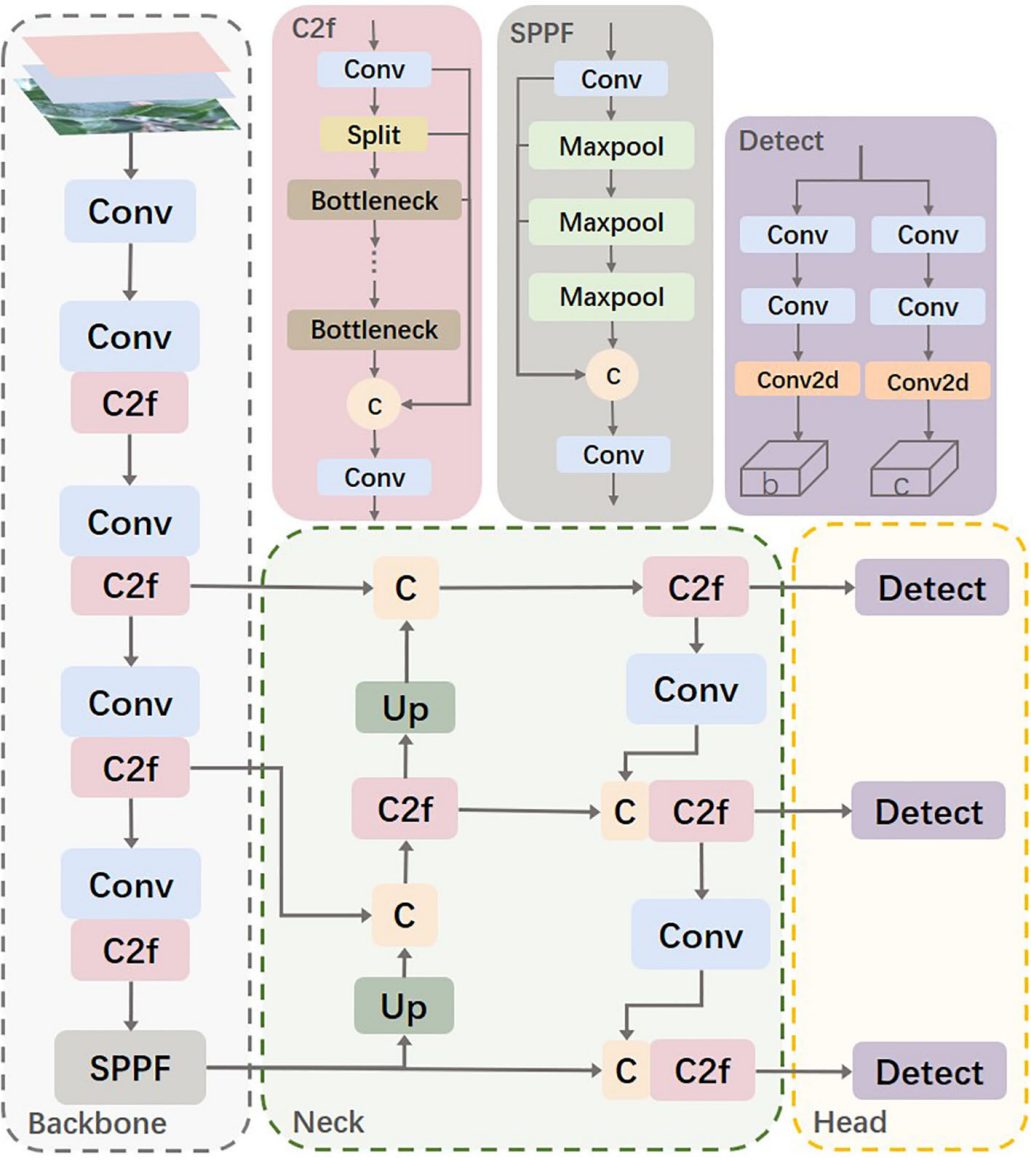
Schematic diagram of the YOLOv8s model.

The YOLOv8s network is similar to YOLOv5, consisting mainly of the Backbone, Neck, and Head. The Backbone part inherits the CSP (Cross Stage Partial) module concept (Wang et al., 2020) but replaces the C3 module in YOLOv5 with the C2f module, which improves network efficiency and performance through more effective feature fusion and gradient flow transmission. Additionally, YOLOv8 modifies the SPP (Spatial Pyramid Pooling) module (He et al., 2015) into the SPPF (Spatial Pyramid Pooling-Fast) module, making detailed adjustments to different scales of the model, instead of using a unified parameter setting. This strategy significantly enhances model performance. In the Neck part, YOLOv8 simplifies the PAN (Path Aggregation Network) structure (Liu et al., 2018) to reduce computational burden while maintaining effective feature fusion. The Head part adopts the currently popular decoupled head structure, which separates the classification and detection heads. Moreover, it transitions from an anchor-based approach to an anchor-free design, enhancing the model’s ability to adapt to targets of different sizes and shapes. **Figure 2** shows the architecture of the YOLOv8 model.

During network training, the loss function is a tool used to represent the difference between predicted and actual values. It plays a crucial role in the training of disease detection models. In YOLOv8s, multiple loss functions are combined for training bounding box regression, classification, and confidence. The loss functions used are as follows:


(1) $$L_{cls}+L_{loc}=L_{conf}$$

where $L_{cls}$ represents the classification loss, $L_{loc}$ represents the bounding box regression loss, and $L_{conf}$ represents the confidence loss. The classification loss $L_{cls}$ uses Varifocal Loss, which combines Focal Loss and binary cross-entropy (BCE):


(2) $$L_{cls}=-\alpha_{t}{\left(1-p_{t}\right)}^{\text{γ}}y_{t}log\left(p_{t}\right)-\left(1-\alpha_{t}\right)P_{t}^{\text{γ}}\left(1-y_{t}\right)log\left(1-p_{t}\right)$$

where $p_{t}$ is the model’s predicted probability for the correct class, $y_{t}$ is the ground truth label (1 if the sample belongs to the current class, otherwise 0), and $\alpha_{t}$ and γ are scaling factors to control sample weight and focusing degree. The localization loss $L_{loc}$ considers the position and shape of the bounding box:


(3) $$L_{loc}={\text{λ}}_{1}\cdot CIoU\;Loss+{\text{λ}}_{2}\cdot DFL\;Loss$$

where ${\text{λ}}_{1}$ and ${\text{λ}}_{2}$ are hyperparameters that adjust the relative importance of the two losses in the total loss.


(4) $$CIoU=1-IoU+\frac{\rho^{2}\left(b_{\text{center }},b_{\text{center }}^{gt}\right)}{c^{2}}+\alpha \cdot v$$

where represents the Intersection over Union, is the distance between the center points of the predicted box and the ground truth box, c is the diagonal length of the smallest enclosing box covering both the predicted and ground truth boxes, v is the aspect ratio consistency term, and is a proportionality coefficient.


(5) $$DFL=-\sum_{i=1}^{n}w_{i}log\left(p_{i}\right)$$

Where $w_{i}$ is the weight, usually adjusted according to the position of the true bounding box. $p_{i}$ is the probability of each class in the predicted probability distribution.

The confidence loss uses Binary Cross-Entropy:


(6) $$L_{conf}=-\left[y\cdot log\left(\hat{y}\right)+\left(1-y\right)\cdot log\left(1-\hat{y}\right)\right]$$

where y is the ground truth label (1 if there is an object within the bounding box, otherwise 0), and $\hat{y}$ is the model’s predicted confidence, representing the probability of an object being within the bounding box, usually the output processed by a sigmoid activation function.

## 4 Proposed algorithm

**Figure 3:**
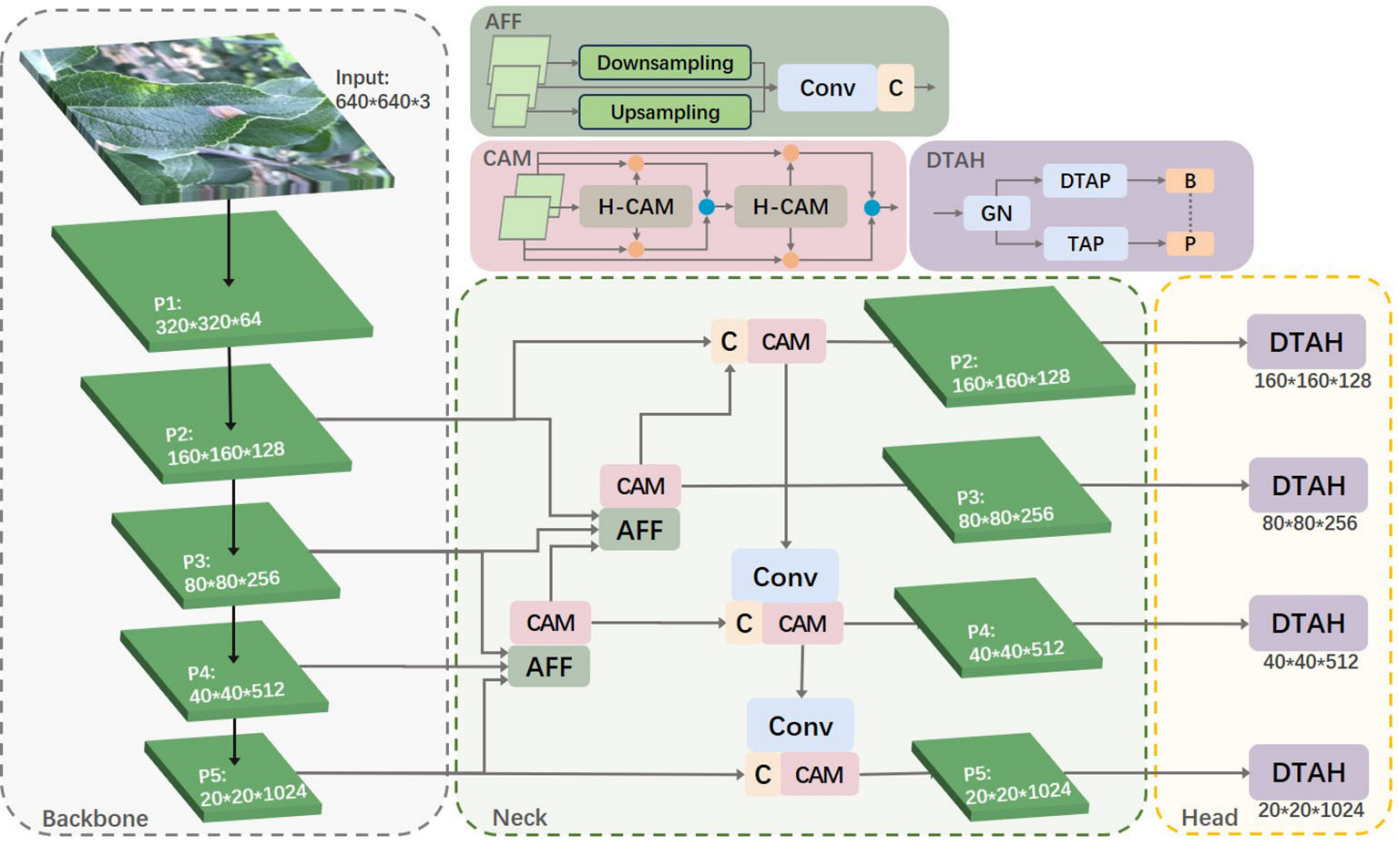
Schematic diagram of the improved model.

Leveraging the strengths of the YOLOv8s algorithm, we propose an enhanced algorithm for identifying apple leaf diseases. This improved algorithm increases the accuracy of disease detection in complex backgrounds while maintaining real-time performance. The enhancements focus on three key aspects: small target feature extraction, cross-layer feature fusion, and task alignment of the detection head. **Figure 3** illustrates the overall framework of the apple leaf disease identification model.

### 4.1 Multilayer feature fusion

YOLOv8s utilizes the PAN-FPN network. In simple terms, the Feature Pyramid Network (FPN) enhances the entire pyramid with top-down transmission of high-level semantic features but only enhances semantic information without passing on localization information. To address this, PAN supplements FPN by adding a bottom-up pyramid after FPN, allowing strong localization features from lower levels to be transmitted upwards. This approach further enhances the multi-scale feature representation, making PAN perform exceptionally well in object detection tasks. When the stride of the backbone network is set to 2, the network increases the downsampling ratio, thereby obtaining richer semantic information. This information is crucial for understanding the extensive context and structure of the target, significantly improving the detection capability of the overall object. However, in this study, the diseases of interest are mostly captured in real outdoor environments, with each image containing multiple targets of varying sizes, even within the same class. The increased downsampling ratio can lead to the loss of a substantial amount of detailed feature information. The output layer of the YOLOv8s object detection model only fuses features from the P3, P4, and P5 layers. Therefore, we considered utilizing the features from the P2 layer to enhance small object detection capabilities while expanding the model’s receptive field. The specific idea is to fuse the P2 layer features with other layers and design the P2 layer as a separate small object detection layer, as shown in **Figure 3**. This approach not only strengthens the model’s ability to detect small targets but also avoids overfitting caused by the model’s excessive reliance on features of a particular size.

**Figure 4:**
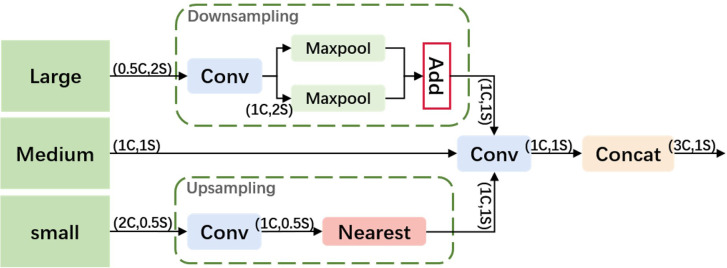
The Adjacent Feature Fusion.

To better utilize shallow features, we designed an Adjacent Feature Fusion (AFF) module, as shown in **Figure 4**. This module cascades information from large, medium, and small layers, providing clearer and richer feature information, especially in complex backgrounds or densely overlapping objects. The module first adjusts the channel numbers of feature maps of various scales to be consistent with the main scale features.

**Large Feature Map Processing:** A convolution module adjusts the channel number of the large feature map to 1C to reduce computational complexity. It employs a hybrid structure of max pooling and average pooling for down-sampling, aiming to reduce the spatial dimension of features while retaining key information, thereby enhancing the model’s translation invariance and adaptability to spatial changes in the input image.

**Small Feature Map Processing:** Similarly, the channel number is first adjusted through a convolution module. Nearest neighbor interpolation is used for up-sampling to retain more local feature details, which is crucial for densely overlapping small objects. This interpolation method leverages adjacent pixel information to minimize feature information loss.

**Feature Map Fusion:** The processed large, medium, and small feature maps are first convolved along the channel dimension and then concatenated. This method fully integrates information of different scales, enhancing the expressiveness of features. The output feature map has the same resolution as the medium-scale feature map, and the channel number is tripled, ensuring that the information from different scale features is effectively fused and strengthened.

### 4.2 Cascade attention module

**Figure 5:**
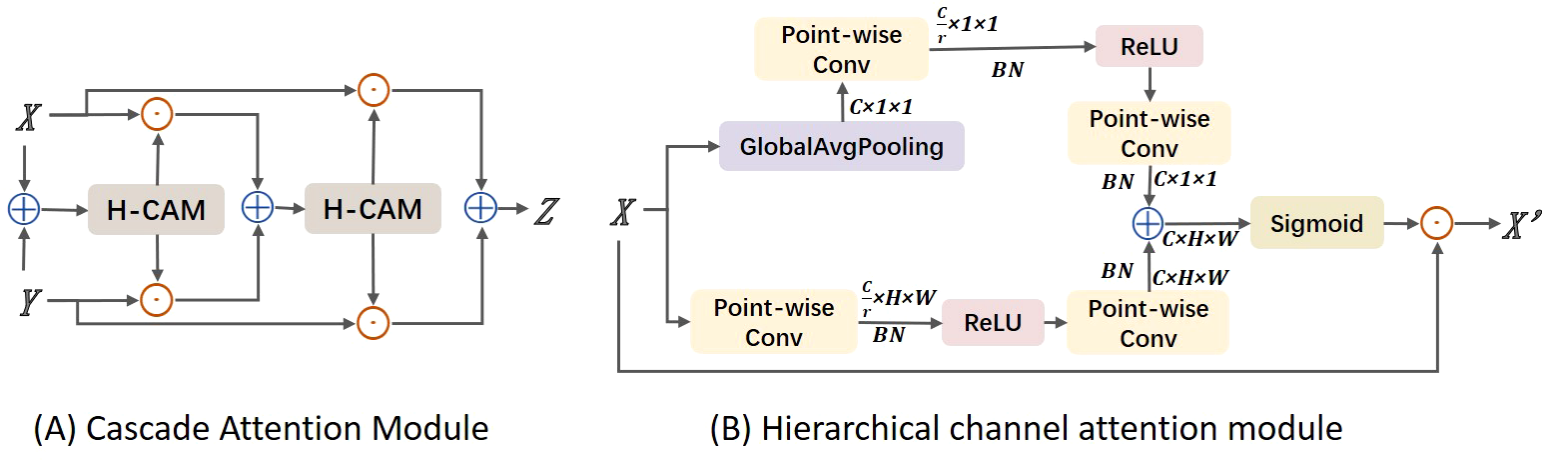
Structure diagram of cascade attention module. **(A)** Cascade Attention Module; **(B)** Hierarchical channel attention module.

In this study, images often contain multiple leaves and complex backgrounds, such as branches and fruits. To address this issue, we considered ways to enhance the model’s ability to localize disease regions, distinguishing target areas from background areas. At the same time, we could not overlook previously mentioned challenges such as target dispersion and varying sizes. Therefore, we considered using adjacent layer feature fusion while incorporating attention mechanisms to strengthen target localization. However, traditional global channel attention mechanisms, such as those used in SKNet and ResNeSt, primarily focus on soft feature selection within the same layer and do not address cross-layer fusion. They also tend to aggregate global information, which can weaken the features of small objects. This is because these methods overly emphasize global context while neglecting the scale differences of objects of various sizes. To address the aforementioned issues, we propose a Cascaded Attention Mechanism feature fusion module (CAM), as shown in **Figure 5A**. CAM utilizes a Hierarchical Channel Attention Module (H-CAM) for refined feature processing, as illustrated in **Figure 5B**, to better capture the relationships between features during the fusion process. H-cam extracts multi-scale channel attention features by combining global pooling and local convolution methods. Specifically, the H-cam module consists of two components: global context and local context. Global context is obtained through Global Average Pooling (GAP), while local context is extracted using Point-wise Convolution. These two contextual pieces of information are then used to generate attention weights.

In implementation, CAM initially performs feature fusion through simple addition, then adjusts the fused features using attention weights generated by H-cam to improve fusion accuracy. The specific calculation formula is as follows:


(7) $$F_{fused}=M(F_{1}⊕F_{2})\;⊗F_{1}+\;(1\;-H_{cam}(F_{1}⊕F_{2}))\;⊗F_{2}$$

Among them, $F_{fused}$ represents the fused feature, $F_{1}$ and $F_{2}$ are the two input features, $H_{cam}$ represents the attention weights generated by H-CAM, ⊕ denotes the initial feature fusion operation (such as addition or concatenation), and ⊗ represents element-wise multiplication. The core of H-CAM lies in its multi-scale channel attention mechanism, which is implemented through the following steps:

**Global Context Extraction:** Perform global average pooling on the input features. The value at the *i*-th row and *j*-th column of the *c*-th channel, $X_{c,i,j}$ is averaged to obtain the global pooling result $G{\left(X\right)}_{c}$ for the *c* -th channel. By averaging all elements of each channel, the global feature vector G(X) is obtained.

**Local Context Extraction:** Perform two point-wise convolution operations *W*, two batch normalization operations *BN* and a ReLU function *δ* on the input features to extract local context features, resulting in the local context feature *L*(*X*).

**Multi-scale Attention Weight Generation:** Add the global and local context features, pass them through a ReLU function and a point-wise convolution, and then generate the multi-scale attention weights *M*(*X*) through a sigmoid function.

The specific formula is as follows:


(8) $$L\left(X\right)=BN_{2}\left(W_{2}\times \delta \left(BN_{1}\left(W_{1}\times X\right)\right)\right)$$

(9) $$G{\left(X\right)}_{c}=\frac{1}{H\times M}\sum_{i=1}^{H}\sum_{j=1}^{W}X_{c,i,j}$$

(10) M(X)=σ(L(X)+G(X))

### 4.3 Dynamic task-aligned head

Object detection is typically formulated as a multi-task learning problem by jointly optimizing object classification and localization. The classification task is designed to learn distinctive features focused on the key or prominent parts of objects, while the localization task is used to precisely locate the entire object with its boundaries. In the domain of disease detection, the accuracy of both tasks is indispensable. The YOLOv8s detection head structure adopts the mainstream decoupled head (Ge et al., 2021), where classification and localization operate in parallel. In the context of apple leaf diseases, the localization task, facing complex and irregular disease features, requires the ability to adapt to local variations in the data. However, when the classification task runs parallel to the localization task, there can be discrepancies in the spatial distribution of learning features between the two tasks. Using two separate branches for prediction can lead to a certain degree of misalignment, resulting in lower detection accuracy.

**Figure 6:**
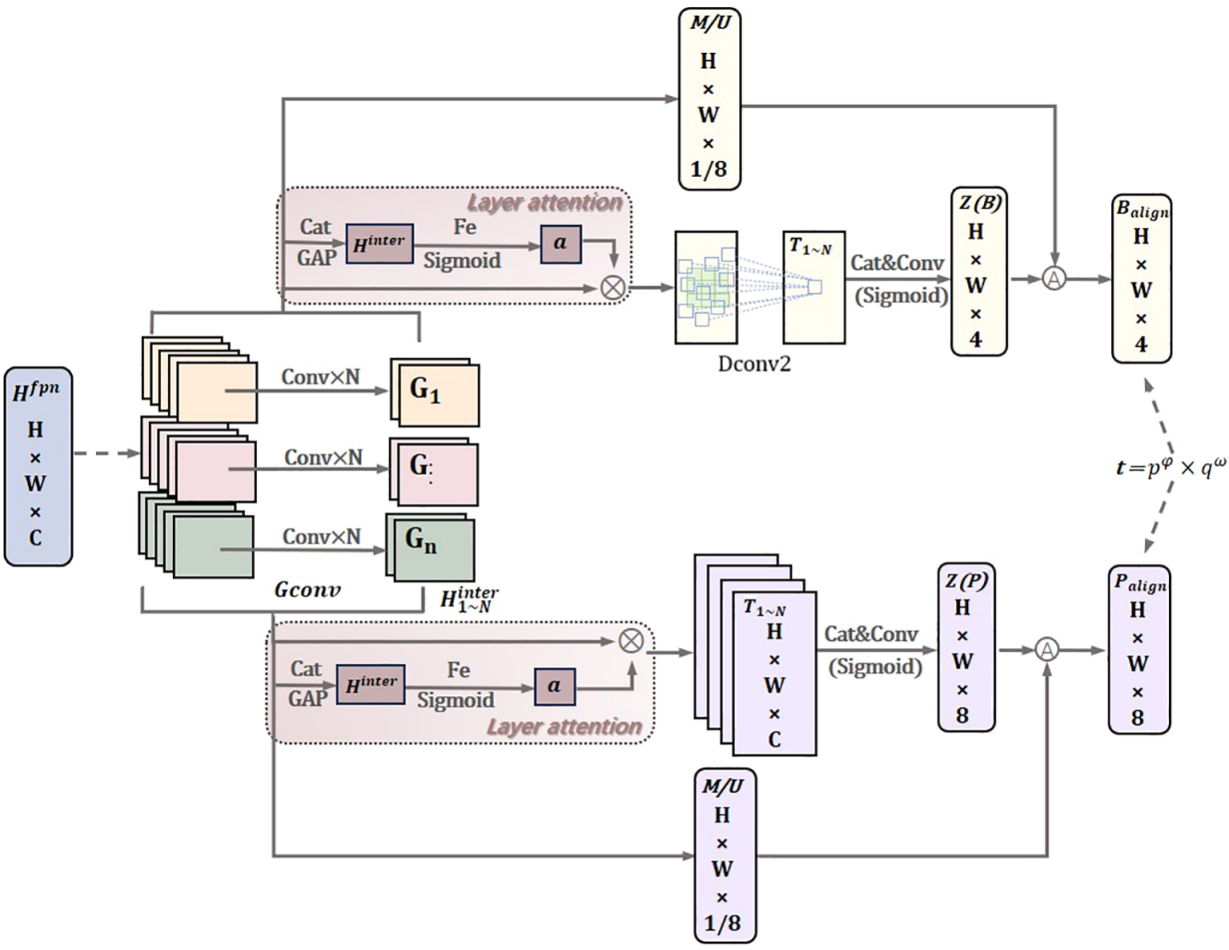
Dynamic task-aligned head.

To enhance the model’s generalization ability and address the misalignment issue between the two tasks, we propose the DTAH head structure, as shown in **Figure 6**. We address the above problems by considering the following three points: (1) enhancing the alignment learning capability of the two detectors; (2) using Deformable Convolution in the localization task branch; (3) increasing interaction between the two tasks.

As shown in **Figure 6**, it features a simple feature extractor with two Task-Aligned Predictor branches (TAP/DTAP). To enhance the interaction between classification and localization while controlling the number of model parameters, grouped convolution is employed to learn task interaction features from multiple convolutional layers. This design not only controls the model size and facilitates task interaction but also provides multi-level features with multi-scale effective receptive fields for both tasks. Here, H, W, and C represent the height, width, and number of channels, respectively. The feature extractor uses N consecutive convolutional layers with activation functions to compute task interaction features:


(11) $$H_{k}^{inter}=\{\begin{array}{l} \delta \left(conv_{k}\left(H^{fpn}\right)\right),k=1\\ \delta \left(conv_{k}\left(H_{k-1}^{inter}\right)\right),k>1 \end{array},\forall k\in \left\{1,2,\ldots ,N\right\}$$

Among them, $conv_{k}$ and *δ* refer to the *k*-th convolutional layer and the ReLU function, respectively. Therefore, we use a single branch in the head to extract rich multi-scale features from the FPN features. The computed task interaction features are then fed into the two task-aligned branches for aligning classification and localization.

**Figure 7:**
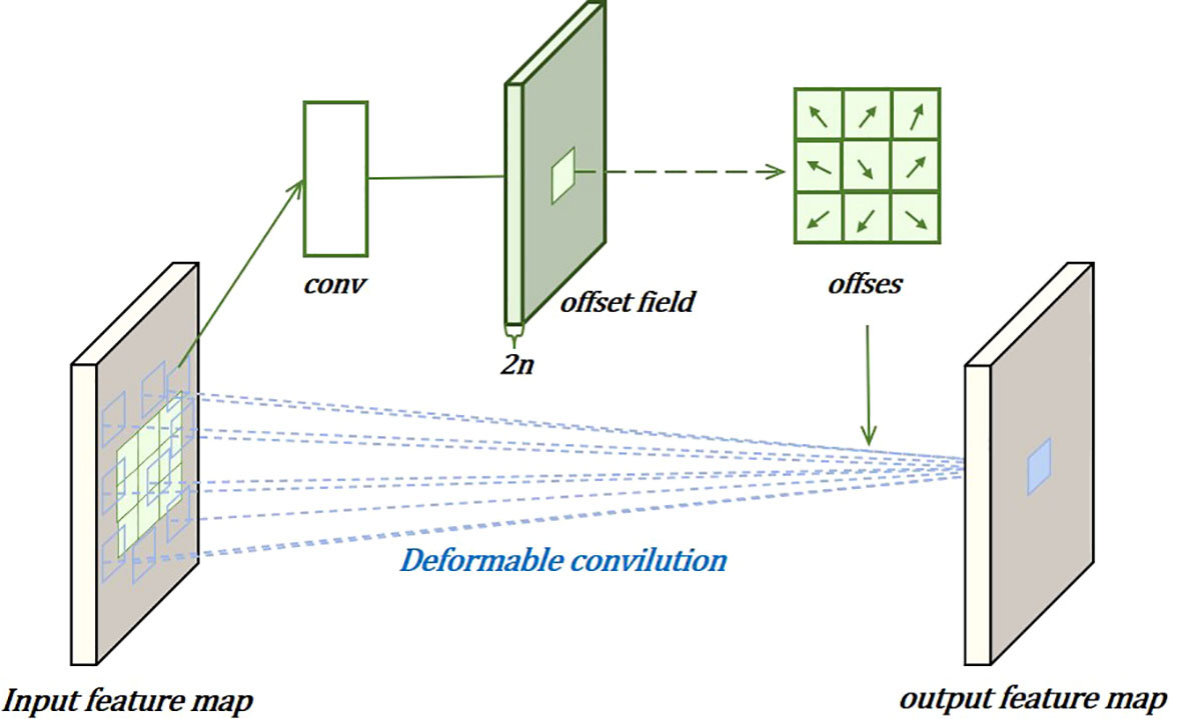
Deformable conv module.


In the localization task, to more effectively capture complex and irregular feature information, deformable convolutions are used to dynamically adjust the convolution kernels, improving localization performance, as shown in **Figure 7**. This involves adding learnable parameters $\Delta P_{n}$ . Similarly, for each output $y\left(p_{0}\right)$ , nine positions are sampled from *x*. These nine positions are obtained by spreading out from the central position $x\left(p_{0}\right)$ , but with the added $\Delta p_{n}$ , allowing the sampling points to spread into a non-grid shape.

(12) $$Y_{P_{0}}=\underset{P_{n}\in R}{\sum }w_{p_{n}}\cdot x\left(P_{0}+P_{n}+\text{Δ}P_{n}\right)$$

Here, $w_{p_{n}}$ is the weight, *x* is the input feature layer, and $\text{Δ}P_{n}$ is the offset learned by convolving the original feature layer, used to adjust the sampling positions of the convolution kernel. Perform object classification and localization on the computed task interaction features, allowing the two tasks to effectively perceive each other’s states. The use of the layer attention mechanism aims to avoid functional conflicts caused by the different objectives of the object classification and localization tasks. This is achieved by dynamically computing task-specific features at the layer level, encouraging task decomposition. Task-specific features for each classification or localization task are calculated separately as follows:


(13) $$T_{k}=a_{k}\cdot H_{k}^{inter},\forall k\in \left\{1,2,\ldots ,N\right\}$$

Among them, $a_{k}$ is the *k*-th element of the learned layer attention $a\in R^{N}$ . The attention *a* is computed from the cross-layer task interaction features and is capable of capturing dependencies between layers:


(14) $$a=\sigma \left(c_{2}\left(\delta \left(c_{1}\left(H^{inter}\right)\right)\right)\right)$$

Among them, $c_{1}$ and $c_{2}$ refer to the two fully connected layers. σ is the sigmoid function, and $H^{inter}$ is obtained by applying average pooling to $H_{1∼N}^{inter}$ , which is the concatenated feature of. Finally, the classification or localization results are predicted from each *T*:


(15) $$Z=conv_{2}\left(\delta \left(conv_{1}\left(T\right)\right)\right)$$

Among them, *T* is the concatenated feature of $T_{1∼N}$ , and $conv_{1}$ is a 1×1 convolutional layer used for dimensionality reduction. The sigmoid function is then used to convert *Z* into dense classification scores $P\in R^{H\times W\times 8}$ , or to process object bounding boxes through the distance-to-bbox $B\in R^{H\times W\times 4}$ transformation.

In the prediction step, the computed task interaction features are used to jointly consider the two tasks, applying the alignment method to each task separately. A spatial probability map $M\in R^{H\times W\times 1}$ is used to adjust the classification predictions:


(16) $$P_{align}=\sqrt{P\times M}$$

Among them, *M* is computed from the interaction features, allowing it to learn the degree of consistency between the two tasks at each spatial location. Simultaneously, to achieve alignment in localization predictions, a spatial offset map $O\in R^{H\times W\times 8}$ is learned from the interaction features to adjust the predicted bounding boxes at each location. Specifically, the learned spatial offset enables the most aligned anchor points to identify the best boundary predictions around them:


(17) $$B_{align}=B\left(i+U_{ij}^{2c},j+U_{ij}^{2c+1},c\right)$$

Among them, (i,j,c) represents the spatial location (i,j) in the *c*-th channel of the *O* tensor. The aligned bounding box $B_{align}$ is achieved through bilinear interpolation, and due to the very small channel dimension of *B*, its computational overhead is negligible. The offset for each channel is learned independently, making the prediction of the four boundaries more precise, as each can independently learn from its most accurate nearby anchor points.

The alignment maps *M* and *U*, which are automatically learned from the stack of interaction features:


(18) $$M=\sigma \left(conv_{2}\left(\delta \left(conv_{1}\left(H^{inter}\right)\right)\right)\right)$$

(19) $$U=conv_{4}\left(\delta \left(conv_{3}\left(H^{inter}\right)\right)\right)$$

Among them, $conv_{1}$ and $conv_{3}$ are two 1×1 convolutional layers used for dimensionality reduction.

Within the framework of Task-Aligned Learning, the learning of *M* and *U* is executed through a dynamic sample allocation strategy, which selects high-quality anchors based on predefined criteria. This selection involves not only the allocation of anchors but also the weighted processing of these anchors. To effectively address the challenges of Non-maximum Suppression (NMS), the allocation of anchors should follow these rules: First, well-aligned anchors should be able to jointly predict objects with high localization accuracy and high classification scores; second, misaligned anchors should have low classification scores and should be suppressed in subsequent processes.

Based on these considerations, we designed a new anchor alignment metric to explicitly evaluate the task alignment degree of each anchor. This alignment metric takes into account both the classification score and the Intersection over Union (IoU) between the predicted and actual bounding boxes, which together indicate the quality of the task prediction. Specifically, we calculate the anchor-level alignment degree for each instance by combining the classification score and the high-order combination of IoU:


(20) $$t=p^{\varphi }\times q^{\omega }$$

Among them, *p* and *q* represent the classification score and the IoU value, respectively. The parameters φ and ω are used to control the influence of the two tasks in the anchor alignment metric. From the perspective of joint optimization, *t* encourages the network to dynamically focus on task-aligned anchors, playing a crucial role in achieving the alignment of the two tasks.

## 5 Experimental details

### 5.1 Experimental setup

In this study, we used PyTorch with a GPU (Graphics Processing Unit) to build the leaf disease detection model. The experiments utilized the SGD (Stochastic Gradient Descent) optimizer, and the details of the hardware and software configurations are shown in **Table 2**. Based on experience from previous related studies and considering the performance of the equipment, we set the training to 150 epochs, with a batch size of 16 and an input image size of 640×640 pixels. Other parameters were kept at their default values.

**Table 2:** Experimental environment information.

Item	Type
CPU	Intel(R) Core(TM) i7-9700 CPU @ 3.00GHz 3.00 GHz
Ram	16.0GB
GPU	NVIDIA GeForce RTX 3090
Operating System	Windows 11
Cuda	CUDA 13.0
programming language	Python 3.8
Deep learning Frame	PyTorch 1.11.0

### 5.2 Experimental data

**Figure 8:**
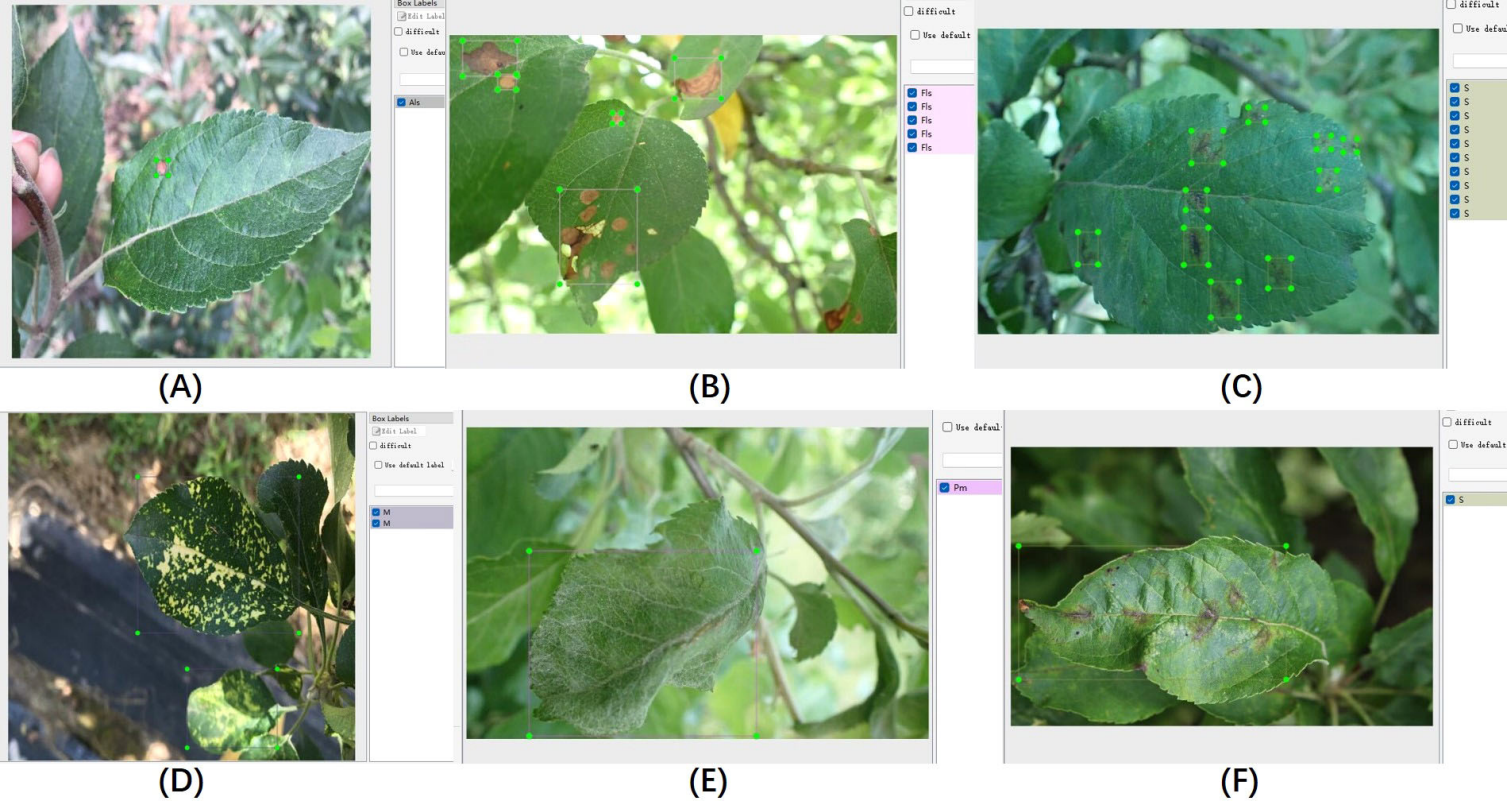
Illustration of the data annotation process. **(A–F)** Display annotations for different disease conditions.

The proposed algorithm was validated on the ALDD dataset, which includes eight common apple leaf diseases captured in either laboratory settings or complex outdoor environments. The dataset consists of 3,638 disease images, and the lesions on the images were annotated using the LabelImg tool under the guidance of domain experts. **Figure 8** illustrates the annotation process. As shown in **Figure 8A**, distinct disease targets were annotated individually. However, when disease features overlapped, as seen in **Figure 8B**, it was decided to annotate them as a whole to prevent missed annotations. **Figures 8D, E** show disease manifestations that cover entire leaves, prompting us to annotate the entire leaf. **Figures 8C, F** display different manifestations of the same disease; we decided to annotate the dispersed form separately while annotating the form that invades the leaf veins or entire leaf as a whole. After annotation, we randomly allocated 80% of each category’s data for training and the remaining 20% for testing. **Table 3** shows the number of training and testing samples for each category, along with their corresponding label names.

**Table 3:** Annotation names and quantities of apple diseases.

Category	Train	Test	Sum	Label	Instances
Alternaria leaf spot	307	77	384	Als	682
Brown spot	463	116	579	Bs	690
Frogeye leaf spot	330	82	412	Fls	830
Grey spot	300	75	375	Gs	741
Mosaic	478	119	597	M	695
Powdery mildew	276	69	345	Pm	557
Rust	292	73	365	R	722
Scab	465	116	581	S	814
Sum	2911	727	3638		

### 5.3 Evaluation metrics

To select the optimal model, the experiments used metrics such as Average Precision (AP), Recall (R), and Mean Average Precision (mAP) to evaluate the performance of the object detection model. R represents the proportion of true positive cases correctly identified by the model out of all actual positive cases, indicating the model’s ability to retrieve relevant instances. AP is calculated as the area under the Precision-Recall curve at various thresholds. mAP is the average of the AP values for all categories and serves as a measure of the overall performance of the object detection algorithm.

Parameter count (Params) refers to the number of trainable parameters in a neural network model. Frames Per Second (FPS) is a measure of the model’s processing speed, indicating the number of image frames the model can handle per second.

The evaluation metric formulas are as follows:


(21) $$Precision=\frac{TP}{TP+FP}$$

(22) $$Recall=\frac{TP}{TP+FN}$$

(23) $$AP=\int_{1}^{0}p\left(r\right)\;dr$$

(24) $$mAP=\frac{1}{m}\sum_{i=1}^{m}AP_{i}$$

(25) $$FPS=\frac{1}{T_{frame}}$$

In the formulas:

**TP (True Positive)** refers to the number of correctly identified positive samples. This means the model correctly classifies actual positive cases as positive.

**TN (True Negative)** refers to the number of correctly identified negative samples, meaning the model correctly classifies actual negative cases as negative.

**FP (False Positive)** represents the number of negative samples incorrectly identified as positive, indicating the model incorrectly predicts actual negative cases as positive.

**FN (False Negative)** refers to the number of positive samples incorrectly identified as negative, meaning the model incorrectly predicts actual positive cases as negative.

In addition, p(r) represents the function relationship where Precision changes with Recall, which can be used to plot the Precision-Recall curve. The variable *m* denotes the number of classes, which equals 8 in this study. The variable *T_frame_* indicates the processing time per frame.

## 6 Experimental results

### 6.1 Ablation study results

To verify the impact of each improvement module in the proposed YOLO-ACT algorithm on the performance of apple leaf disease detection, we individually integrated the AFF, CAM, and DTAH modules and evaluated the model performance on the ALDD dataset, maintaining the same training environment as previously described. When all improvement modules work synergistically, the model’s convergence speed significantly increases. The training and testing loss function curves are detailed in **Supplementary Figure S1**.

**Figure 9:**
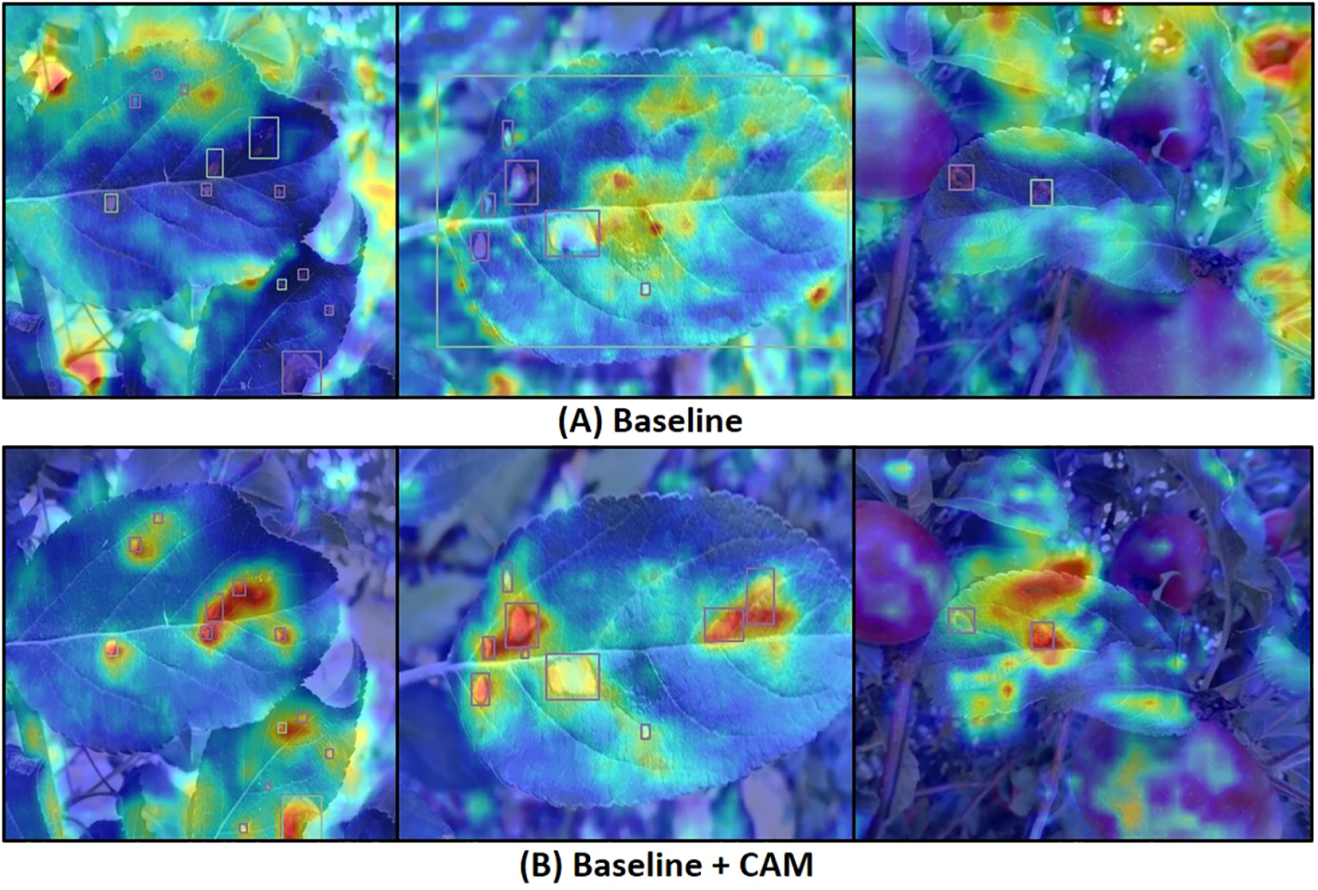
Add CAM heatmaps comparison. **(A)** baseline; **(B)** Baseline + CAM.

**Table 4** presents the average precision (AP), recall (R), and mean average precision (mAP) metrics after incorporating each improvement module. The results indicate that the mAP improved from 82.3% to 83.7% after adding the AFF module, demonstrating the module’s significant role in enhancing feature extraction and fusion. To investigate the effectiveness of the CAM attention module, we generated heatmaps via channel activation to observe the attention mechanism’s focus areas in disease images. **Figure 9** compares heatmaps before and after incorporating the attention module, with red indicating regions of primary focus, yellow as secondary, and blue as redundant areas. It is evident that the model exhibits higher activation values in diseased regions after integrating the attention mechanism, indicating that the attention mechanism improves the model’s focus on important features while ignoring redundant areas, leading to more accurate decision-making. The mAP increased by another 0.7% when CAM and AFF worked together, further confirming the CAM module’s effectiveness. Finally, after adding the DTAH module, the model achieved the highest performance metrics, with an AP of 84.4%, R of 78.6%, mAP of 85.1%, and mAP@[.50:.95] of 58.3%. This indicates that the DTAH module enhances the interaction between classification and localization tasks, thereby improving the model’s detection performance. The line charts visualizing the training process are shown in **Supplementary Figure S2**, clearly illustrating that each improvement module proposed in this study enhances YOLOv8’s performance in apple leaf disease detection. Additionally, the final algorithm demonstrates a noticeably faster convergence speed compared to the baseline.

**Table 4:** Annotation names and quantities of apple diseases.

Baseline	AFF	CAM	DTAH	AP(%)	R(%)	mAP(%)	mAP50-95(%)
✓				79.3	75.3	82.3	56.1
✓	✓			83.9	76.1	83.7	56.7
✓		✓		83.0	76.9	83.2	56.5
✓			✓	82.5	76.6	84.0	56.7
✓	✓	✓		83.9	78.3	84.4	57.1
✓	✓		✓	81.7	78.0	84.5	57.4
✓		✓	✓	83.8	77.1	84.1	56.8
✓	✓	✓	✓	84.4	78.6	85.1	58.3

### 6.2 Comparison of experimental results

To evaluate the performance of our proposed YOLO-ACT algorithm, this study compares it with several classic algorithms. We utilized the same dataset, training settings, and evaluation criteria across all models to ensure a fair comparison. The specific comparison results are as follows: **Table 5** presents the results of various versions of the YOLO series algorithms. **Table 6** shows the mAP of eight different disease categories on the ALDD dataset for the YOLO series algorithms.

**Table 5:** Comparison of detection results of YOLO series classic algorithms on ALDD.

Method	AP(%)	R(%)	mAP(%)	mAP50-95(%)	Params(m)	FPS
Yolov5s	83.6	76.4	79.1	54.2	7.1	103
Yolov6s	80.5	69.0	78.5	50.5	17.9	68
Yolov7-tini	74.8	73.4	76.6	46.8	6.0	112
Yolov9s	82.0	76.0	83.7	56.9	9.6	40
Yolov10s	81.1	76.3	82.9	54.2	8.1	94
Yolov8s	79.3	75.3	82.3	56.3	11.1	117
Ours	84.4	78.6	85.1	58.3	9.9	76

**Table 6:** mAP for eight disease categories on ALDD.

Label	Yolov5s	Yolov6s	Yolov7-tini	Yolov8s	Yolov9s	Yolov10s	Ours
Als	80.6	71.7	82.7	78.5	83.6	79.8	**84.3**
Bs	72.3	83.4	65.8	83.1	87.2	**87.6**	86.9
Fls	87.0	82.8	87.6	89.9	89.2	89.4	**90.4**
Gs	74.5	74.8	80.8	76.2	79.3	79.9	**80.8**
M	69.1	73.9	80.4	80.0	**81.1**	78.4	78.6
Pm	64.6	68.1	64.2	72.1	75.5	74.0	**75.7**
R	89.1	87.6	90.5	89.9	91.8	88.0	**92.0**
S	**95.5**	86.0	61.2	89.0	82.3	85.8	92.3

The values in bold indicate the highest mAP achieved by each label across various models.

The comparative experimental results indicate that after incorporating the three improvement modules, our proposed YOLO-ACT algorithm shows a trade-off in terms of parameter count and frame rate but demonstrates significant advantages in mAP and related metrics for apple leaf disease detection. Compared to the baseline, our algorithm improved mAP by 2.8%, with increases of 5.1%, 3.3%, and 2% in AP, R, and mAP50-95, respectively. Among the compared algorithms, including YOLOv5s, YOLOv6s (Li et al., 2022), YOLOv7-tiny (Wang et al., 2023), YOLOv8s, YOLOv9s (Wang et al., 2024b), and YOLOv10s (Wang et al., 2024a), our model achieved the highest mAP. Compared to the latest YOLOv10s, our model’s mAP is 2.2% higher.

In this experiment, Mosaic and Powdery mildew are the most complex categories, with all models showing suboptimal detection accuracy, with the best mAP around 80%. This is due to their high intra-class variability: the colors can be light or dark, and the disease symptoms do not follow traditional spot patterns. Additionally, the high similarity between Alternaria leaf spot and Gray spot makes them difficult to distinguish. On the other hand, the symptoms caused by Rust and Apple scab are relatively consistent, and their lesion shapes are distinct from other types, making them easier to identify visually. Consequently, all models maintain high detection accuracy for these diseases. Specifically, our model achieved the highest detection accuracy in Alternaria leaf spot, Frog-eye leaf spot, Gray spot, Powdery mildew, and Rust, with mAPs of 84.3%, 90.4%, 80.8%, 75.7%, and 92.0%, respectively. These results indicate that the improvement modules (AFF, CAM, DTAH) introduced in our model significantly enhance its feature extraction and fusion capabilities, improving the model’s adaptability in handling complex backgrounds and multi-scale objects. Moreover, YOLOv5 performed well in distinguishing Apple scab, achieving a 95.5% mAP, while our model achieved over 90% accuracy in detecting multiple diseases. Overall, our YOLO-ACT model not only excels in detection accuracy but also demonstrates superior robustness and adaptability, providing a more reliable and efficient solution for disease detection in apple cultivation.

**Figure 10:**
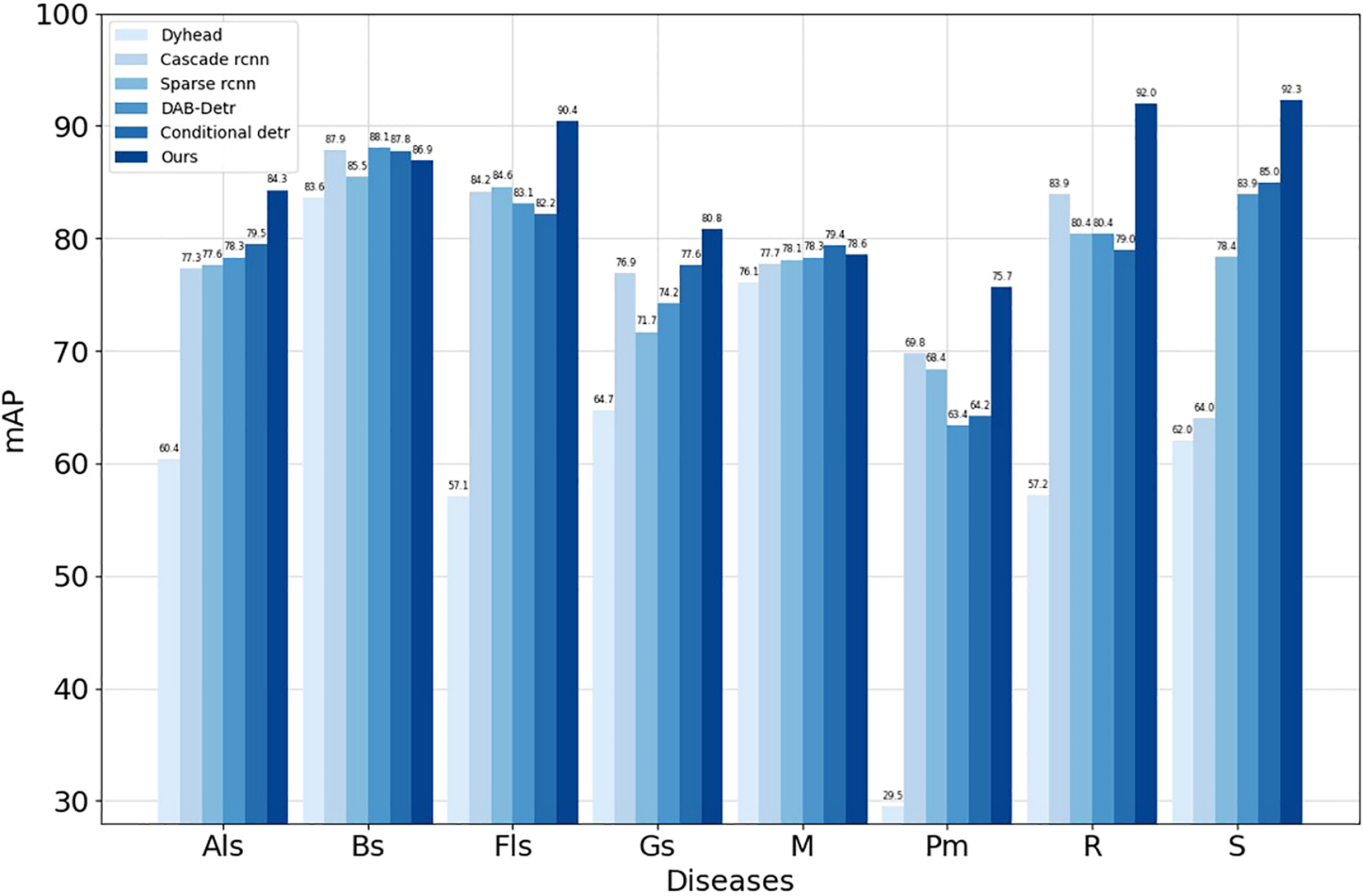
Comparison of mAP for eight disease categories identified by different algorithms.

To further demonstrate the effectiveness of our algorithm in apple leaf disease detection, we trained second-stage and transformer advanced algorithms using the MMDetection toolbox, with the results for each metric shown in **Table 7**. Compared to the Dynamic Head (Dai et al., 2021), Cascade R-CNN (Cai and Vasconcelos, 2018), Sparse R-CNN (Sun et al., 2021), DAB-Detr (Liu et al., 2022), and Conditional Detr (Meng et al., 2021) algorithms, our algorithm not only achieves the highest mAP but also meets the requirements for mobile devices in terms of parameter quantity and frame rate, making it suitable for mobile devices. From the per-category mAP in **Figure 10**, our research algorithm achieves the highest average precision in six categories: Glomerella leaf spot, Frog eye leaf spot, Gray mold, Powdery mildew, Rust, and Black spot. The other two categories also perform well, demonstrating the effectiveness of our research algorithm.

**Table 7:** Results of other advanced algorithms on ALDD.

Method	AP(%)	R(%)	mAP(%)	Params(m)	FPS
Dyhead	61.1	40.7	61.3	38.9	30
Cascade R-CNN	77.9	60.2	77.8	69.4	66
Sparse R-CNN	78.1	59.6	78.1	42.4	35
DAB-Detr	78.8	66.4	78.8	43.7	19
Conditional Detr	79.5	63.7	79.5	43.45	45
Ours	84.4	78.6	85.1	9.9	60

The results clearly indicate that while our proposed algorithm is less lightweight compared to other algorithms, it still meets the requirements for real-time detection and excels in detection capabilities, ranking first among all compared algorithms. However, we observed a common pattern across all experimental data: R is consistently lower than AP. This suggests that when dealing with leaf disease data in complex backgrounds, the model tends to ignore background interference and classify uncertain areas as background to reduce false positives. Therefore, selecting results with higher confidence is more advantageous for leaf disease detection in complex backgrounds.

### 6.3 Visualization and discussion

**Figure 11:**
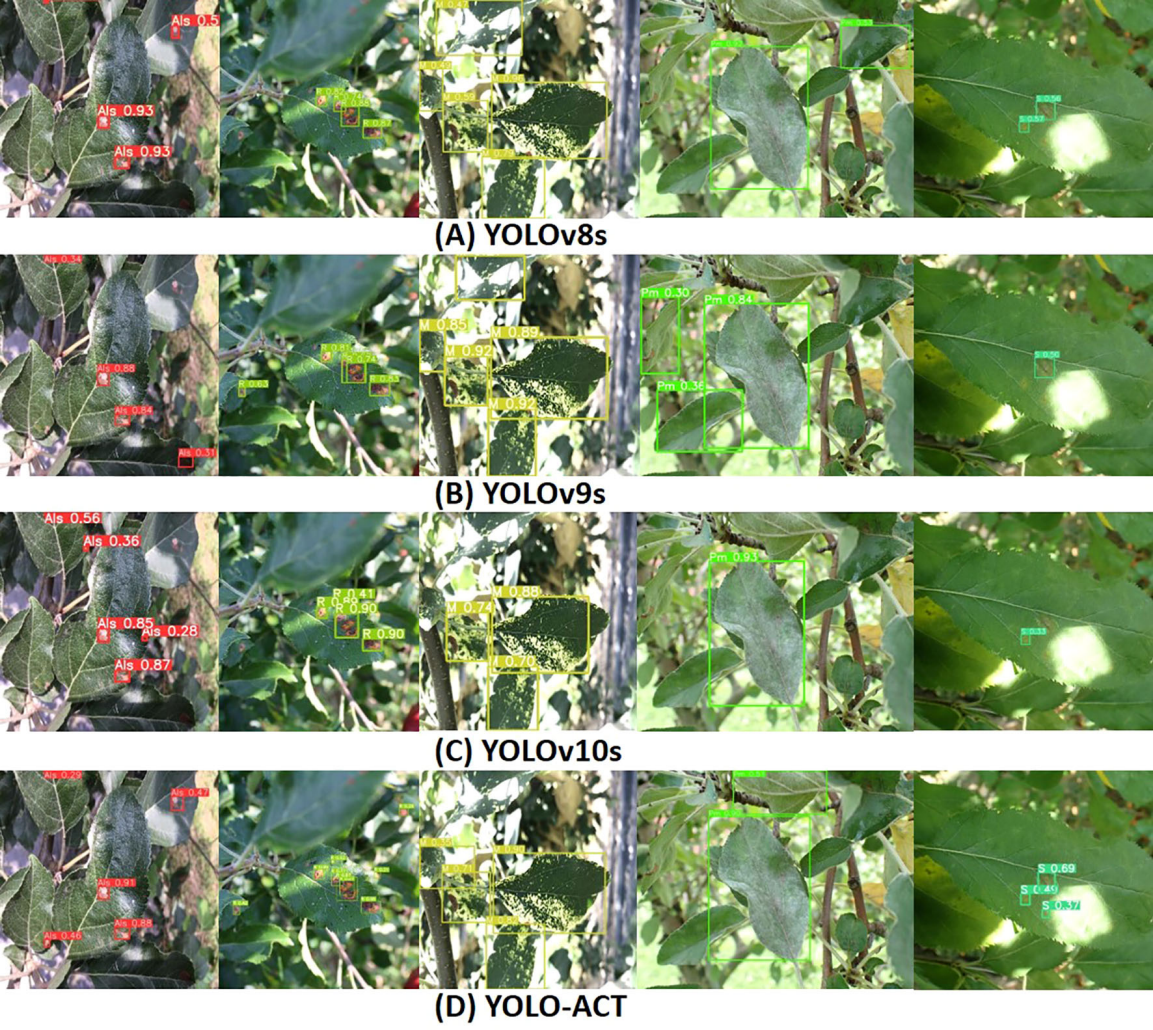
Visualization of detection results on ALDD. **(A)** YOLOv8s; **(B)** YOLOv9s; **(C)** YOLOv10s; **(D)** YOLO-ACT.

**Figure 11** illustrates the visual detection results of the latest YOLO series algorithms on the ALDD dataset, compared with our proposed algorithm and baseline models. The first three rows in **Figure 11** showcase the detection performance of YOLOv8s, YOLOv9s, and YOLOv10s under complex background conditions, while **Figure 11D** displays the performance of our proposed algorithm under the same conditions. In the first column, YOLOv8s and YOLOv9s exhibit significant missed detections due to the tendency to overlook extremely small targets in such complex backgrounds. The second column shows that YOLOv10s also has missed detections, indicating variability in detection performance across different diseases. In the third column, strong lighting interferes with the models’ detection of mosaic disease, with YOLOv8s and YOLOv9s showing better detection results under these conditions. The fourth column illustrates the detection results for powdery mildew, where all models perform poorly. Specifically, YOLOv8s is misled by the strong light, resulting in the misclassification of healthy leaves as diseased. In the fifth column, lighting interference in the scab disease areas causes other algorithms to miss the disease. This highlights that complex backgrounds and lighting conditions increase the demand for model generalization capabilities in disease localization. However, YOLO-ACT can still make accurate judgments under these conditions. In summary, YOLO-ACT demonstrates superior performance in detecting apple leaf diseases in natural environments, regardless of the interference from complex surroundings or the difficulty in recognizing disease characteristics.

**Figure 12:**
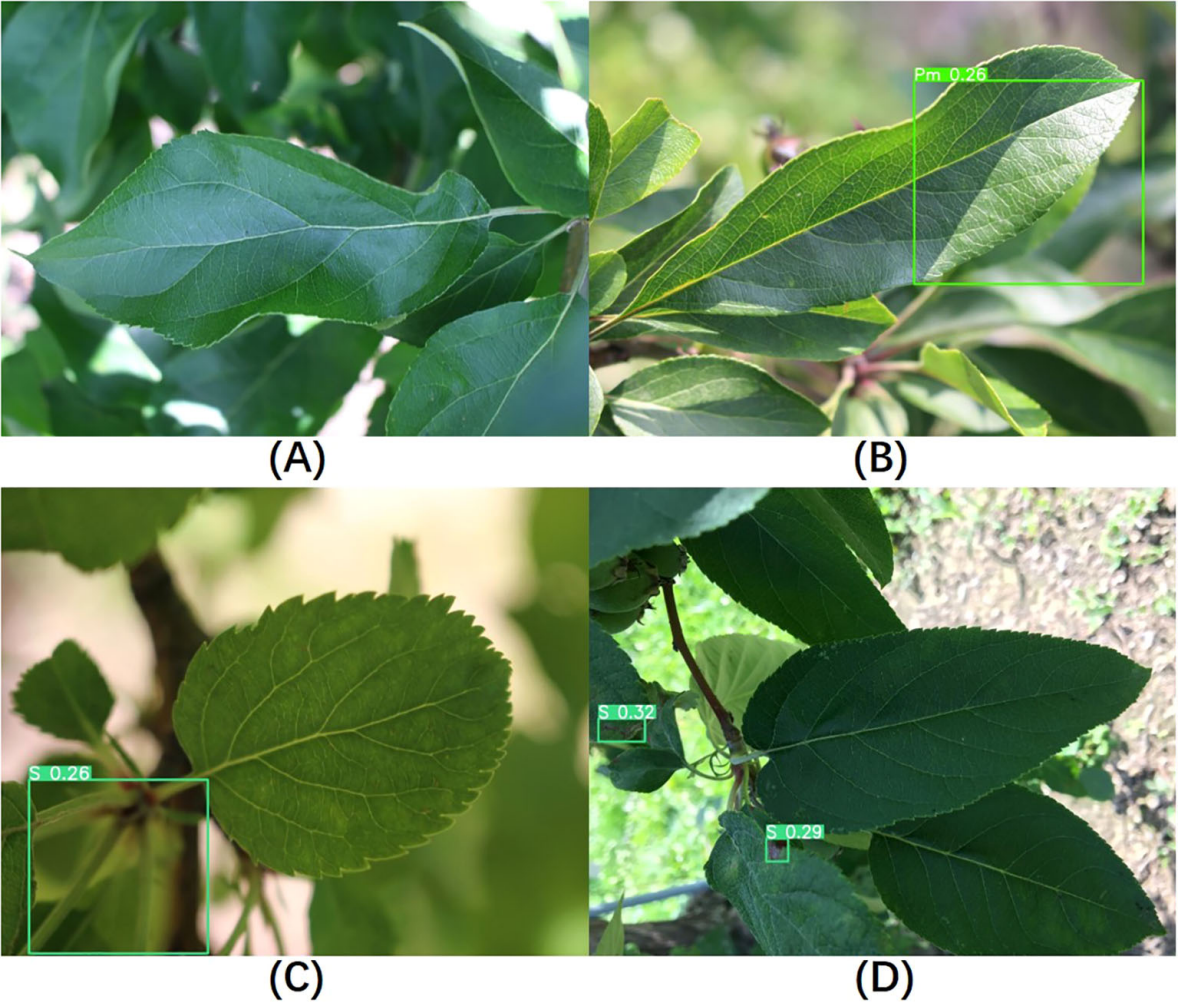
Visualization of healthy leaf detection results. **(A–D)** Different results observed when detecting healthy leaves.

Since this study focuses on the localization and classification of diseases on leaves, healthy leaves without diseases were not included in the training process. However, to evaluate the model’s performance on healthy leaves, we randomly selected 300 healthy leaf images for testing. Among them, 233 images showed no significant changes, indicating the absence of disease in the images, as shown in **Figure 12A**. We classified these as correct results. In the remaining 67 images where diseases were detected, three different outcomes were observed. The first type of false detection, shown in **Figure 12B**, occurred due to light reflections; even with very low confidence level, the model still framed healthy areas. The second type of false detection, illustrated in **Figure 12C**, involved the model mistakenly identifying tangled petioles as disease areas. In the third scenario, shown in **Figure 12D**, the model successfully identified the main leaf in the image as a non-disease area, but it framed and classified disease regions on other leaves in the background of the image. We meticulously reviewed these images and discussed the classification results with domain experts, manually assessing the model’s classification outcomes. Correct classifications were counted as correct results, while incorrect classifications were considered false detections. Ultimately, the testing accuracy for these 300 healthy leaf images was 87.7%. Using the same approach to test YOLOv8s yielded an accuracy of 83.7%, indicating that our model has also improved its performance in distinguishing healthy leaves compared to the baseline model.

## 7 Conclusion

In this study, we proposed an improved detection algorithm named YOLO-ACT to enhance the accuracy of apple leaf disease detection in complex backgrounds. We integrated the AFF cross-layer feature fusion module and a small target detection layer into the Neck of YOLOv8s, which improved the model’s ability to extract features from small targets. The C2f module was replaced with the CAM module featuring a cascaded attention mechanism, which, in conjunction with AFF, significantly enhanced feature fusion capabilities. The addition of the DTAH detection head improved task interaction and alignment, leading to enhanced model performance. On the ALDD dataset, our model achieved an AP of 84.4%, a Recall of 78.6%, and an mAP of 85.1%, outperforming YOLOv5s, YOLOv6s, YOLOv7-tiny, YOLOv8s, YOLOv9s, and YOLOv10s, thus demonstrating its effectiveness.

The model’s parameter count is 9.9 m with a frame rate of 76 FPS, making it suitable for deployment on mobile platforms, enabling intelligent perception, early warning, decision-making, analysis, and expert online guidance in agricultural environments. However, some challenges remain; results indicate that lighting significantly affects detection accuracy. While the improvements enhanced mAP, they also led to a reduction in FPS. Future work will aim to further improve detection capabilities for apple leaf diseases while also focusing on model speed and size, with an exploration of deployment on mobile devices.

## Data Availability

Publicly available datasets were analyzed in this study. This data can be found here: https://github.com/JasonYangCode/AppleLeaf9.git https://github.com/pratikkayal/PlantDoc-Object-Detection-Dataset.git.
