# Supplementary material for: YOLO-ACT: an adaptive cross-layer integration method for apple leaf disease detection
Source: Front Plant Sci. 2024 Oct 1;15:1451078. doi: 10.3389/fpls.2024.1451078 (PMC11473324; doi:10.3389/fpls.2024.1451078)
Supplement: Supplementary file 1 [file DataSheet1.zip › Supplementary material.DOCX]

**Supplementary material**

**
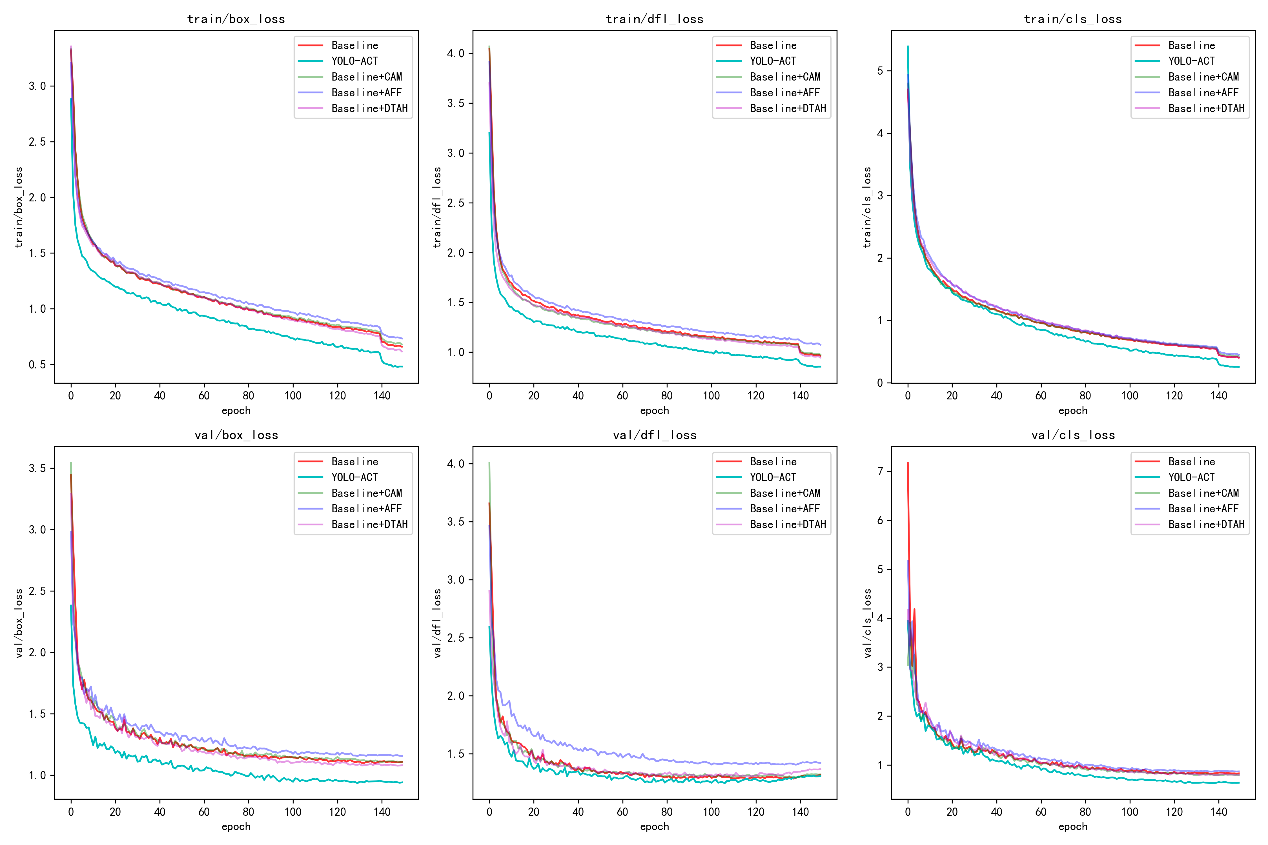
**

**Supplementary Figure S1**. The curve of training and testing loss function

**
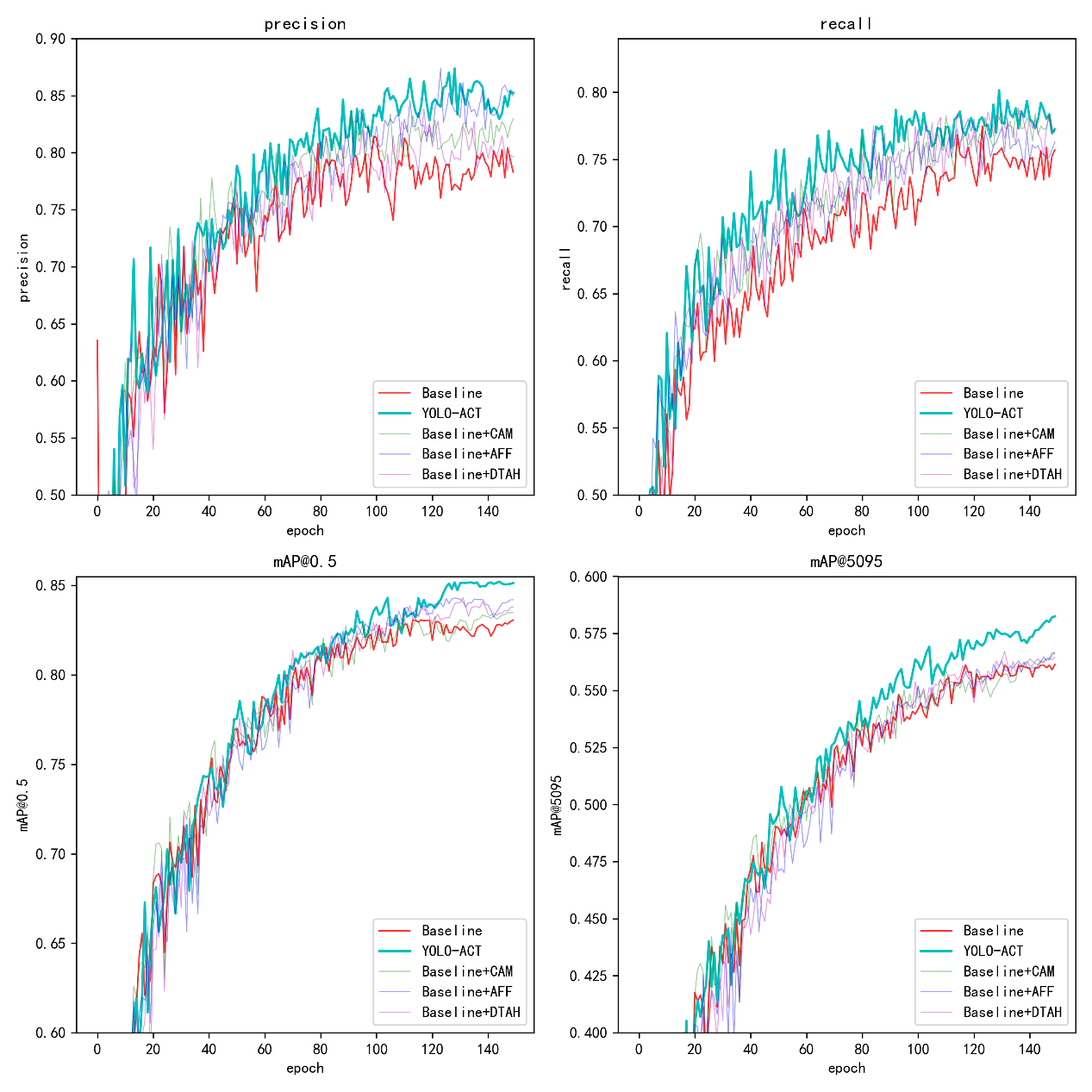
**

**Supplementary Figure S2**. Detection algorithm mAP curve
